# 侧群细胞与肺癌肿瘤干细胞

**DOI:** 10.3779/j.issn.1009-3419.2012.03.10

**Published:** 2012-03-20

**Affiliations:** 300052 天津，天津医科大学总医院，天津市肺癌研究所，天津市肺癌转移与肿瘤微环境重点实验室 Tianjin Key Laboratory of Lung Cancer Metastasis and Tumor Microenvironment, Tianjin Lung Cancer Institute, Tianjin Medical University General Hospital, Tianjin 300052, China

**Keywords:** 肺肿瘤, 侧群细胞, ABCG2/Bcrp1, 肿瘤干细胞, Lung neoplasms, Side population cells, ABCG2/Bcrp1, Tumor stem cells

## Abstract

侧群（side population, SP）细胞是一小群能够将染料Hoechst 33342排出从而在流式细胞图上呈现Hoechst 33342低染的细胞，研究者已在多种肿瘤组织和细胞系中检测出了SP细胞并验证这些SP细胞具有强干细胞活性。现有试验结果证实多种肺癌组织和肺癌肿瘤细胞系中也存在具有类干细胞特性的SP细胞，这些SP细胞的鉴别与分离对于肺癌肿瘤干细胞的研究有何意义？本文将整合现有研究资料就这一问题进行阶段性综述。

## Abstract (en)

Side population (SP) cells are a rare subset of cells, which can be isolated by dual-wavelength flow cytometry because of their capacity to efflux Hoechst 33342, found in various tissues and cell lines that are highly enriched for stem cell activity. As recent studies have identified stem-cell-like SP cells in many lung cancer tissues and cell lines, this review is intended to find out the relationship between lung cancer SP cells and lung cancer stem cells.

Goodell等^[1]^在研究小鼠骨髓细胞中造血干细胞的特性时发现这样一群细胞：当以荧光染料Hoechst 33342标记时，双波长二维流式细胞散点图上会出现一小群Hoechst 33342低染的细胞，试验发现，与整体骨髓细胞相比，这群细胞富集了造血干细胞活性的细胞，他们命名这部分细胞为侧群细胞，即SP（side population）细胞。之后，在许多组织器官的干细胞和肿瘤细胞中都检测出了含量很少的SP细胞，这些SP细胞也都相应地具备干细胞的多种特性，被认为是一个浓缩的干细胞群体^[2-6]^。2005年，Kim等从支气管和肺泡导管交界处分离出了支气管肺泡干细胞（bronchial alveolar stem cells, BASCs），BASCs表现出明显的自我更新特性，能够分化为其它类型的肺上皮细胞，当体内支气管和肺泡损伤时，BASCs能够分化为终末细支气管上皮细胞（Clara细胞）、Ⅰ型肺泡上皮细胞和Ⅱ型肺泡上皮细胞，研究^[7]^还发现，*K-ras*基因突变活化可引起BASCs增殖，提示BASCs可能是肺腺癌的起源细胞，这一研究结果更是激起了人们探寻肺癌肿瘤干细胞的兴趣。2007年，Ho等^[8]^从包括A549在内的6种人肺癌细胞系和肿瘤组织中分离出了SP细胞，并试验验证了这些SP细胞具有类干细胞特性，提出采用SP表型可以分离纯化得到肺癌肿瘤起源性细胞。目前分离肿瘤干细胞的方法主要包括根据特异性分子标记物分选和根据SP细胞表型进行分选^[9]^，而肺癌肿瘤干细胞的特异性表面标记尚未得到确认，肺癌SP细胞的分离和鉴定很有可能为肺癌肿瘤干细胞的研究提供便利的途径，本文将就肺癌SP细胞对于研究肺癌肿瘤干细胞的意义进行阶段性综述。

## 1 肺癌SP细胞的干细胞特性验证

Patrawala等^[3]^总结出干细胞的本质特性包括：①通常所占比例都很低；②尽管具有很强的繁衍潜能，但在正常微环境中基本不分化；③能实现自我复制；④具有多向分化的能力；⑤能表达特殊标记物或生物特征以实现识别与富集。近来研究者已先后在A549、H460、H23、H441、H2170、HTB-58、H446和SPC-A1等多种人肺癌细胞株中分选得到了SP细胞，它们占总细胞比例的1.1%-6.3%，并且90%以上都处于G_0_期-G_1_期。体外培养发现，这些肺癌SP细胞能够以非对称方式分化产生SP细胞与非SP细胞，且分化增殖后SP细胞占总细胞的比例与分选前原细胞中SP细胞比例相近，非SP细胞则只能增殖而不能分化产生SP细胞。裸鼠接种试验^[8, 10-12]^结果表明，与非SP细胞和整体细胞相比，接种SP细胞的成瘤率提高了几十倍甚至几百倍。对从A549细胞中分选出的SP细胞进行microRNA分析，发现其表达谱与非SP细胞有着明显差异，SP细胞的microRNA表达丰度要远低于非SP细胞，这符合干细胞microRNA的表达随着分化的进行而逐渐增高的结论^[13]^。

## 2 ABCG2/Bcrp1对研究肺癌SP细胞和肺癌肿瘤干细胞的意义

现有研究^[2, 4, 14]^已证实，细胞排出染料Hoechst 33342从而表现出SP表型的能力与ABC转运蛋白家族有关，特别是*ABCG2*/*Bcrp1*基因与SP表型直接相关，在Bcrp1^-/-^小鼠的骨髓细胞中，SP表型缺失。ABCG2/Bcrp1基因是导致细胞在传统化疗过程中产生多药耐药性的主要转运子之一，与肿瘤干细胞的强抗药性特征相符，那么该基因是否可以作为肺癌肿瘤干细胞的特异性标志物呢？试验结果发现，在Bcrp1^-/-^小鼠的骨髓细胞中，虽然SP表型缺失，但是造血干细胞的数量与功能仍然维系在正常水平，这一发现提示，即使Bcrp1能够作为HSCs的一个标记物，但对其干细胞的功能并无直接影响^[14]^。Patrawala等^[3]^通过对30多种肿瘤细胞系进行反复试验验证，得出如下结论：虽然肿瘤细胞中的SP细胞群较非SP细胞群成瘤性强，但ABCG2^+^细胞和ABCG2^-^细胞的成瘤性并无明显差异。Zhou等^[15]^报道了关于小鼠肺组织中的SP细胞分布和Bcrp1表达的研究，研究结果指出，虽然SP细胞高表达Bcrp1，但并不是所有表达Bcrp1的细胞都具有SP表型。牛等^[16]^也通过免疫组化方法观察了ABCG2在肺癌组织及肺癌细胞系GLC-82和A549中的表达，发现在大细胞和小细胞癌组织中基本未检出ABCG2，而在肺腺癌和肺鳞癌组织中ABCG2呈弥漫性阳性分布，在两株细胞系中ABCG2也几乎100%阳性表达，可见ABCG2/Bcrp1虽然与细胞的SP表型直接相关，但是单独并不能作为肺癌肿瘤干细胞的筛选标记。

此外，朱等^[17]^的另一发现对于研究ABC转运蛋白与SP表型以及SP表型细胞的高肿瘤干细胞特性之间的关系也很有意义，他们在SPC-A1/Docetaxel细胞中检测出了较SPC-A1细胞更高比例的SP细胞，但前者中的SP细胞在增殖能力、克隆形成能力以及致瘤性方面都比后者中的SP细胞要低，他们分析认为，前者细胞中的高比例SP细胞主要是由于经过了药物筛选从而高表达ABC转运蛋白所致，而非全部由天然高表达ABC转运蛋白的肿瘤干细胞产生。由此提示，并不是任何情况下得到的SP表型细胞都能成为肿瘤干细胞的富集。

## 3 肺癌肿瘤干细胞标志物的研究现状

CD133是细胞表面蛋白超家族中的一员，已在乳腺癌、脑胶质瘤、肝癌、结肠癌和前列腺癌等多种实体瘤中被用作肿瘤干细胞标志物^[9]^。Eramo等^[18]^从小细胞和非小细胞肺癌组织和细胞系中分离得到了一小部分含量极少的CD133^+^细胞，通过试验他们发现，这些细胞具有自我复制和多向分化的能力，在无血清培养基中能够无限增殖，当以10^4^数量级对免疫缺陷小鼠进行接种时即能发展成为同型瘤体，他们认为这部分未分化的肺癌CD133^+^细胞就是肺癌的肿瘤源性细胞。而Meng等^[19]^的研究结果显示，肺腺癌细胞A549和小细胞肺癌细胞H446中的CD133^+^细胞和CD133^-^细胞中肺癌肿瘤干细胞含量相似，它们无论在体外克隆、细胞的自我更新和分化，还是体内成瘤和侵袭，以及对化疗药物的耐药性等各方面的实验结果并无明显差异。钱等^[20]^的研究结果也发现，CD133^+^细胞在人肺腺癌组织、癌旁组织和正常肺组织中广泛分布，其数量在三者之间无统计学差异。

越来越多的研究^[9]^发现，以CD133为代表的单个标记物应用于肺癌肿瘤干细胞的分离与鉴别具有较大的局限性，而多个表面标记物联合则有助于提高鉴别的特异性及灵敏度。例如，Kim等^[7]^发现肺腺癌可能的起源细胞BASCs表达Sca-1^+^CD45^-^PECAM^-^CD34^+^，Levina等^[21]^将肺癌细胞株H460通过阿霉素、顺铂、依托泊苷处理后得到的药物生存细胞（drug surviving cells, DSCs）培养后能形成新的克隆，试验鉴定这些DSCs具有高成瘤高转移等肿瘤干细胞特性，它们除了高表达CD133、CD117和OCT-4等干细胞标记物，还高表达VEGF、bFGF、IL-6、IL-8和受体VEGFR2、FGFR2等。Gutova等^[22, 23]^则应用干细胞标记物CD34、CD90、CD133、ABCG2/Bcrp1来分离小细胞肺癌肿瘤干细胞。

## 4 小结

由上文的综述可见，关于肺癌肿瘤干细胞的特异性标志物到目前为止还没有得到统一的确认，虽然*ABCG2*/*Bcrp1*是决定细胞SP表型的重要基因，但并不是表达该基因的肺癌细胞都具有SP表型，它既不能直接影响肺癌SP细胞的干细胞活性，也不能单独作为肺癌肿瘤干细胞的标志物。同样，也并不是所有的肺癌SP细胞都是肺癌肿瘤干细胞。Challen等^[2]^提出，在缺乏确定的细胞表面标志的情况下，SP表型对于某些组织干细胞而言可能并不是很有效的分离标志，并且从不同器官分离的SP细胞具有异质性，只有在明确的证明单个SP细胞能分化为多个起源于特定组织的细胞时，组织特异性SP细胞才能被认为是干细胞。

不过，由于SP细胞具有广泛性和表型上的保守性，还是很有可能成为肺癌肿瘤干细胞研究的理想模型。以肺癌SP细胞作为研究模型，应用微阵列分析等高通量分子生物信息学手段比较肺癌细胞中的SP细胞与正常肺组织干细胞的SP细胞基因表达的差异，对于寻找肺癌肿瘤干细胞特有的标志物有重要意义。依靠特异性标志物不仅能有效减少甚至杜绝化疗时的严重毒副作用，同时也将为肺癌的早期诊断和预后奠定基础。
